# Compartmentalized Culture of Perivascular Stroma and Endothelial Cells in a Microfluidic Model of the Human Endometrium

**DOI:** 10.1007/s10439-017-1797-5

**Published:** 2017-01-20

**Authors:** Juan S. Gnecco, Virginia Pensabene, David J. Li, Tianbing Ding, Elliot E. Hui, Kaylon L. Bruner-Tran, Kevin G. Osteen

**Affiliations:** 1 Women’s Reproductive Health Research Center, Vanderbilt University Medical Center, Nashville, TN USA; 2 Department of Pathology, Immunology and Microbiology, Vanderbilt University Medical Center, Nashville, TN USA; 3 School of Electronic and Electrical Engineering, University of Leeds, Woodhouse Lane, Leeds, LS2 9JT UK; 4 School of Medicine, Leeds Institute of Biomedical and Clinical Sciences, University of Leeds, Leeds, UK; 5 Department of Biomedical Engineering, University of California, Irvine, CA USA; 6 Veteran Affairs Tennessee Valley Healthcare System, Nashville, TN USA

**Keywords:** Endometrium, Stroma, Organs-on-a-chip, Microfluidic, Porous membrane

## Abstract

The endometrium is the inner lining of the uterus. Following specific cyclic hormonal stimulation, endometrial stromal fibroblasts (stroma) and vascular endothelial cells exhibit morphological and biochemical changes to support embryo implantation and regulate vascular function, respectively. Herein, we integrated a resin-based porous membrane in a dual chamber microfluidic device in polydimethylsiloxane that allows long term *in vitro* co-culture of human endometrial stromal and endothelial cells. This transparent, 2-μm porous membrane separates the two chambers, allows for the diffusion of small molecules and enables high resolution bright field and fluorescent imaging. Within our primary human co-culture model of stromal and endothelial cells, we simulated the temporal hormone changes occurring during an idealized 28-day menstrual cycle. We observed the successful differentiation of stroma into functional decidual cells, determined by morphology as well as biochemically as measured by increased production of prolactin. By controlling the microfluidic properties of the device, we additionally found that shear stress forces promoted cytoskeleton alignment and tight junction formation in the endothelial layer. Finally, we demonstrated that the endometrial perivascular stroma model was sustainable for up to 4 weeks, remained sensitive to steroids and is suitable for quantitative biochemical analysis. Future utilization of this device will allow the direct evaluation of paracrine and endocrine crosstalk between these two cell types as well as studies of immunological events associated with normal vs. disease-related endometrial microenvironments.

**Electronic supplementary material:**

The online version of this article (doi:10.1007/s10439-017-1797-5) contains supplementary material, which is available to authorized users.

## Introduction

**Figure 1:**
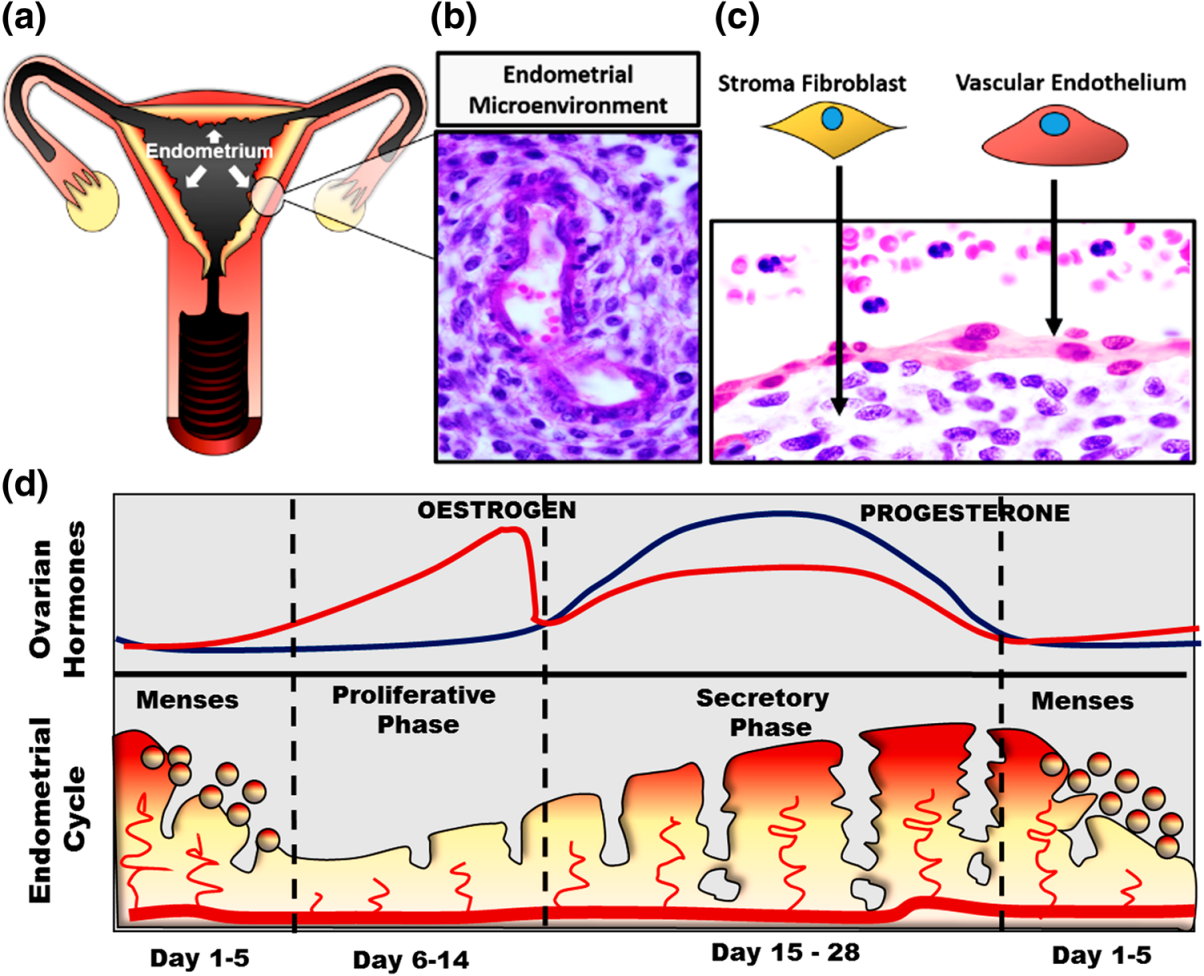
The human endometrium. (a) A schematic representation of the human female reproductive tract. (b) A histological image of the endometrial micro-environment in the secretory phase of the menstrual cycle shows decidualization of the stromal fibroblasts in regions surrounding endothelial vessels (original magnification ×400). (c) False colouring of peripheral leukocytes, and endothelial cells of the endometrial perivascular stroma (original magnification ×1000) (Haematoxylin and eosin staining, in b and c). (d) A schematic summary of the ovarian hormones changes during the endometrial cycle: the menstrual cycle can vary widely among individual women, but an idealized length is generally considered to be 28 days. During each menstrual cycle, the endometrium undergoes a proliferative phase (6–14 days) when oestrogen concentrations rise up to a level capable of triggering ovulation and the subsequent development of the corpus luteum. In the secretory phase (days 15–28), production of ovarian progesterone promotes endometrial differentiation. The endometrium later experiences a sharp withdrawal of ovarian sex steroids, resulting in an inflammatory cascade that leads to the shedding of the endometrial tissue over 3–6 days, a process known as menstruation and which marks the beginning of a new cycle (days 1–6).^16^,^18^–^20^,^39^

The female reproductive tract is composed of interactive organs, including uterus and ovaries that are physiologically regulated by endocrine signals at a spatial–temporal level.^14^ The endometrium lines the inner cavity of the uterus and is the primary maternal tissue responsible for establishing embryo implantation and the successful maintenance of pregnancy.^2^ Somatic cell numbers and immune cell ratios within the endometrium vary significantly across each phase of the menstrual cycle. However, histologic analysis of the cycling endometrium consistently reveals luminal and glandular epithelial cells supported by specialized reticular stromal fibroblasts (stroma), a vascular system and a dynamic flux of immune cells^39^ (Fig. 1).

Endometrial tissue homeostasis, cellular proliferation, metabolism and reproductive function are mediated by the crosstalk between these cell types through paracrine and endocrine pathways.^3^,^9^,^11^,^31^–^33^ The cyclical changes of ovarian sex steroids production, oestrogen and progesterone, dictate timing and functional capabilities of the endometrium to support embryo implantation. These steroids account for the distinct phases of the menstrual cycle by driving cell-specific morphological and biochemical changes (as depicted in Fig. 1d).

In the endometrium of humans and some other primates,^20^,^37^,^42^ during the secretory phase of a non-pregnant menstrual cycle, increasing levels of ovarian progesterone trigger a partial, spontaneous decidualization process within the stroma.

In all women, the morphological and biochemical differentiation of stroma into specialized decidual cells is clinically recognized as being critical for the successful establishment and maintenance of pregnancy to term. Decidualized stroma exhibit an epithelial-like cuboidal morphology and develop the biochemical capacity to release several pro-gestational molecules including prolactin and insulin-like growth factor binding protein-1. Histological evidence of secretory phase decidualization has been observed to originate in those regions directly surrounding the vasculature (Fig. 1b). While this basic, visual observation is well accepted in the field, the degree to which paracrine signalling mechanisms between vascular cells and adjacent specialized fibroblasts drives early decidualization at this specific site has not yet been determined. At present, a lack of appropriate *in vitro* models of human endometrial cells that allow analysis of potential interactions between key cell types necessary to support the successful establishment and maintenance of pregnancy hinders progress in understanding this critically important aspect of reproductive tract function.^2^,^12^

Many researchers, including our group, have demonstrated an important role for stromal-epithelial cross talk in normal endometrial function while dysregulated cell–cell communication is associated with numerous disease processes^11^,^24^,^31^,^40^; however, the interaction between stroma and their adjacent vascular endothelium has received less investigative attention.^1^ While steroid hormone receptors are largely concentrated in endometrial stromal and epithelial cells (Fig. 1d), it is equally true that the proliferative endometrium becomes increasingly thicker due to endometrial vascularization, as spiral arteries grow within the stroma in response to increasing levels of oestrogen. In regards to this biological relationship, Albrecht *et al.* 1 co-cultured human endometrial somatic cells (i.e. epithelial and stromal cells) with myometrial microvascular endothelial cells using a transwell assay and observed an increase in endothelial tube formation and vascular endothelial growth factor production under the influence of oestrogen.^1^ Together with established histological observations, Albrecht’s *in vitro* study strongly suggests that interactions between endothelial and stromal cells occur *via* active paracrine communication.

In relation to the culture system described herein, the possibility to precisely recreate and visualize morphological and functional changes in a microfluidic model will be critical to better understand not only oestrogen action, but also progesterone-directed communication between these specific cell types during the second half of the human menstrual cycle. In addition to the cyclic oestrogen and progesterone mediated events described above, it is also important to note that biologically significant changes also occur in relation to the immunomodulatory function of endothelial cells at the end of the secretory phase of the menstrual cycle.^21^–^23^,^46^

A more physiologic model system of the perivascular stroma should provide not only a better basic knowledge of normal steroid-mediated endometrial function but also holds promise to reveal how altered patterns of cell–cell communication promotes the pathogenesis of diseases, including breakthrough bleeding, infertility, menorrhagia, endometriosis, pregnancy disorders and endometrial cancer.^20^,^21^ Unfortunately, most *in vitro* models fail to mimic the *in vivo* physiological conditions that the endothelium experiences, including bidirectional paracrine crosstalk between cells and hemodynamic forces. Moreover, current *in vitro* models do not allow for high resolution real-time examination of functionally significant morphological changes. Current transwell assays largely enable modelling of specific cell barriers in human organs, such as epithelium^4^ or endothelium,^36^ with minimal capability to mimic and control the hemodynamic flow conditions observed in the vasculature. Several *in vivo* and *in vitro* studies have defined the role of hemodynamic forces in the regulation of vascular function^35^,^47^: once exposed to shear stress from the continuous blood circulation, endothelial cells undergo cytoskeletal remodelling (from cobblestone shaped in static conditions to elongated cell body in the direction of flow in dynamic conditions)^10^ and become functionally different compared to cells cultured in static conditions.^32^,^33^

Multi-compartmental 3D microfluidic cell culture devices, so called “Organs-on-a-Chip” (OoC) have been introduced to address limitations of *in vitro* modelling.^6^,^8^,^9^,^13^,^15^,^25^,^26^,^28^,^30^ These models represent robust compartmentalized, heterogeneous cell culture systems to simulate the physiology and anatomy of human organs and thus should enhance our understanding of *in vivo* mechanisms that are otherwise difficult to study. *Via* microfluidic compartmentalization, the role of specific cell types can be identified as well as the cell-specific effects of biomechanical forces (e.g. shear stress) and chemical (steroid stimulation) cues that are externally introduced and controlled in the system. Key analytical functions of OoC include real-time imaging of a cell culture, maintenance of long term cultures (a minimum of 4 weeks), non-invasive selective staining and the analysis of secretion and metabolism of the individual cell types by sampling spent media from each compartment. Additionally, while commonly used transwell inserts rely on polyester and polycarbonate membranes that are opaque in bright field or may contribute to high auto fluorescence, PDMS technology results a completely transparent apparatus which does not limit high resolution imaging analysis and thus enhances data interpretation. An important advantage of the OoC is the reduction of the total media volumes required for cell culture thus limiting dilution of nutrients and selected test molecules within the system: these conditions may favour the specific study of the paracrine signalling between the different cell types.^45^

To better understand the interactive role of the perivascular stroma in the human endometrium, we have designed and developed a dual chamber microfluidic device that integrates a resin-based microfabricated porous membrane and allows long term co-culture of human primary umbilical vein endothelial cells (HUVECs) and endometrial stroma. With this OoC model, our group and other researchers will be able to accurately examine and analyse morphological and biochemical aspects of the interactive roles of endometrial stromal and endothelial cells under the influence of sex steroid regulation.

## Materials and Methods

### Fabrication of the 1002F Membranes

A transparent, semi-permeable membrane was used in the device to separate the cell types within two microfluidic chambers. The membranes are fabricated by using a photolithographic method^29^,^41^ with biocompatible resin, EPON 1002F (Miller-Stephenson, Sylmar, CA). Briefly, the 1002F resin and a photoinitiator (UVI-9676, Dow Chemical, USA) were dissolved in γ-butyrolactone (GBL, Sigma Aldrich, USA) at a ratio of 10:1:10 by weight. A 100-mm silicon wafer was treated with plasma (2 min, O_2_, 13.3 Pa) and coated with a sacrificial layer of soap (2% Micro-90, International Products, Burlington, NJ, USA). An 8-μm layer of 1002F (diluted 1:1 in GBL) was deposited by spin coating and then soft baked. The wafers were then exposed through a 125-mm chromium–quartz photomask (10 mW/cm^2^, 14 s). After a post-exposure bake, the patterned resist was developed in propylene glycol methyl ether acetate. Each membrane is patterned with a 6.5 × 6.5 mm^2^ array of 2-μm pores enclosed within a 15-mm diameter circle.

### PDMS Layer Design and Fabrication

**Figure 2:**
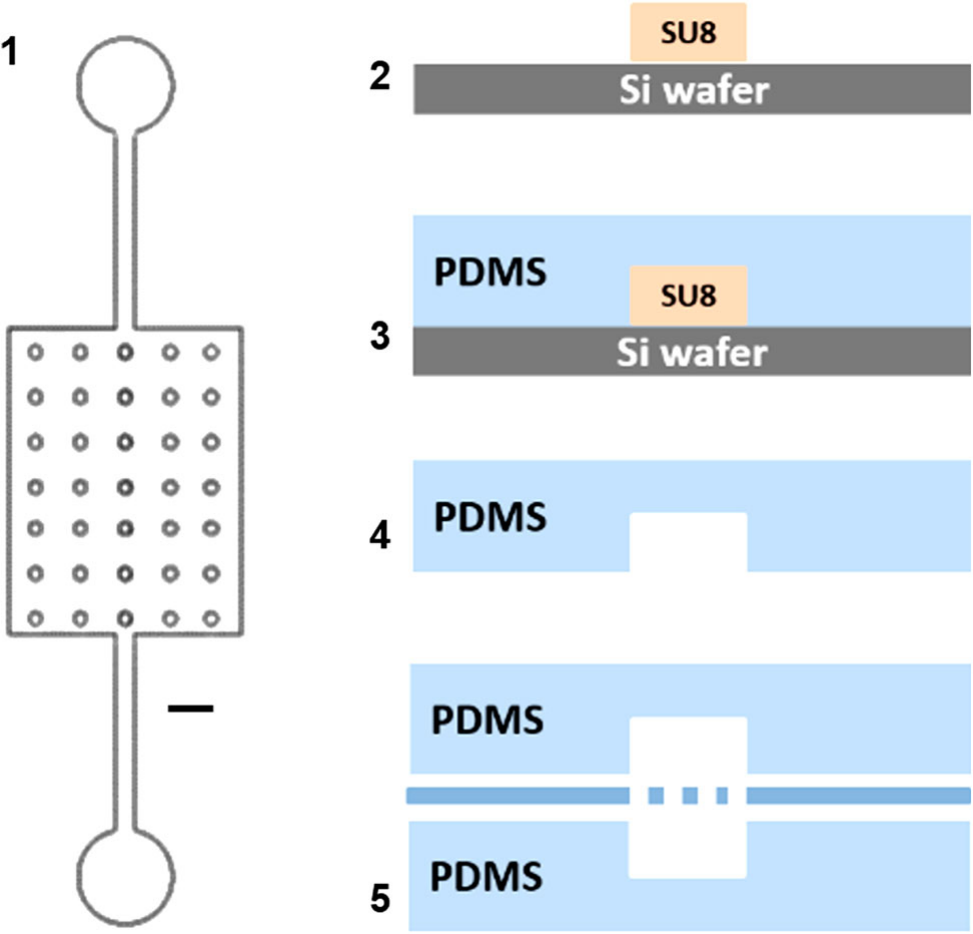
Fabrication process: (1) Mylar mask (40,000 dpi, Infinite Graphics Incorporated, MN, USA): the design for a single chamber includes the 35 circular pillars (diameter 100 μm), scale bar 1 mm. (2) Fabrication of SU 8 master by photolithography: a bas-relief master was fabricated in a Class 100 clean room with SU8-2100 negative photoresist (Microchem, MA, USA). 4″ silicon wafers were spin coated with SU8 (30 s at 2000 rpm, final thickness ~150 μm). The resist layer was exposed to UV light through the mask and finally processed with SU-8 developer to removed unexposed resist. (3) Liquid PDMS (prepolymer to curing agent ratio 10:1) was cast and cured on the SU8 master to obtain two equal layers. (4) Cured PDMS layers were demoulded, and ports to access the microfluidic chambers were opened by punching 1/16″ holes in the top layer with a stainless steel round punch (Integra Myltex, NJ, USA). (5) The porous membrane was plasma activated and sandwiched between to PDMS layers. Soft, post-exposure and final hard baking steps were done following datasheet indications.

The device was assembled using two 4.75 mm by 6.2 mm microfluidic chambers separated by a semi-permeable 1002F membrane. The complete device was fabricated by soft lithography^17^ in polydimethylsiloxane (PDMS, Sylgard^®^ 184, Dow Corning, MI, USA) (Fig. 2) as summarized in Fig. 2.

### Assembly of the Two-Chambers Platform

The 1002F membranes were released from the silicon master by dissolving the sacrificial Micro-90 soap layer. The membranes were carefully removed and inspected for quality prior to bonding. After release, the membranes were kept in sterile H_2_O to remove residual soap and to prevent drying. The top PDMS layer and the 1002F membrane were then oxygen-plasma treated (600 mTorr, 100 W, 45 s) and bonded together. Since both PDMS and 1002F are optically transparent, alignment of various components was completed under a stereomicroscope. The second layer was then bonded with the same method on the 1002F membrane orthogonally to the top PDMS layer.

Oxygen-plasma treatment renders the exposed surfaces hydrophilic. Hence, the assembled devices were immediately filled with sterile DI H_2_O and stored at 4°C until used. For static experiments, 500 μL cloning cylinders (Fisher Scientific, Pittsburgh, PA, USA) were bonded with liquid PDMS to the inlet/outlet regions of each channel to form small reservoirs for the cell media.

### Acquisition of Human Tissues

The Vanderbilt University Institutional Review Board approved the procedures for tissue acquisition, performed only after patients gave informed consent. Primary human umbilical vein endothelial cells (HUVECs) were isolated from umbilical cord,^5^ obtained from de-identified term placenta collected from patients who underwent elective caesarean section between 37 and 39 weeks of gestation. For this phase of establishment, optimization and characterization of the OoC, HUVECS were selected since they represent the most common model based on human derived primary endothelial cells.^34^ After isolation, we consistently observed ≥95% purity of endothelial cells, validated morphologically and by immunofluorescent staining for CD31 (DAKO, USA) before loading in the devices. Cells were cultured in EBM-2 medium supplemented with EGM™-2 Single Quot^®^ growth factors (Lonza, USA), maintained at 37°C in a saturated humidity atmosphere containing 95% air/5% CO_2_, and they were sub-cultured before reaching 60–70% confluence (approximately every 2 days) up to passage 5.

For acquisition of endometrial stroma, surgically excised uterine tissues were collected from consented donors (ages 18–45) exhibiting predictable menstrual cycles and undergoing a hysterectomy for benign leiomyoma not associated with any additional inflammatory ovarian or endometrial disease. Endometrial stroma were isolated by enzymatic digestion and filter separation.^40^ As with our endothelial cell preparation, the purity of the stroma isolation was above 95% and was quantified by morphological assessment and positive staining for vimentin as previously described.^40^ Stroma were maintained in phenol red-free DMEM/F-12 with 10% charcoal-stripped calf serum, 1 nM 17-β oestradiol (Sigma Aldrich, USA) and 1× antibiotic–antimycotic solution (stromal complete growth medium). As required for experimental objectives, some stroma cultures received treatments with 0.5 mM medroxyprogesterone acetate (MPA, Sigma Aldrich, USA) and/or 8-bromoadenosine-3′,5′-cyclic monophosphate (cAMP, 0.5 mM, Sigma Aldrich, USA) to induce a decidualization response over a period of 14 days.

### Cell Culture and Maintenance in Device

Isolated stromal and endometrial cells were initially expanded within 75 mm flasks before transfer to our microfluidic devices. Upon achieving approximately 80% confluent monolayers, both endothelial and stromal cells were separately trypsinized, pelleted by centrifugation (220 rcf), resuspended in full medium (1 × 10^6^ cells/mL) and subsequently loaded into each chamber using a 1 mL syringe. To enhance cell adhesion inside the device we utilized a thin coating of 1:50 dilution of Matrigel (BD Bioscience, USA) on both chambers to provide an extracellular matrix substrate. The cells were allowed to adhere for a minimum of 30 min inside the incubator. 300 μL of EBM-2 medium or complete growth medium, were added to the endothelial top chamber or stromal bottom chamber, respectively. Spent media from the reservoirs were collected and replaced daily with fresh media throughout all static experiments.

For dynamic flow experiments, the endothelial chamber was perfused by using a syringe pump (PicoPlus, Harvard Apparatus, Cambridge, USA) and by connecting Tygon^®^ tubing (Cole-Parmer) directly into the inlet port of the device. Shear stress conditions were induced when the cells reached 60% confluence. The final value of 1 μL/min was defined with this protocol as minimal value for the cells to form a tight and reoriented endothelium. The stroma were maintained under the same static culture conditions through all experiments. Wall shear stress in the center of the chamber (approximately 6 × 10^−3^ dyn s cm^−2^) was calculated as in Sung *et al*.^44^ treating the chamber as a relatively flat channel (with width ≫ height). Bubble formation was limited by loading the microfluidic chambers with DI water immediately after plasma treatment and by equalizing the temperature of the media in the syringe and inside the device before starting the perfusion.

### Fluorescence Imaging

Mouse monoclonal anti human CD-31 primary antibody (DAKO, USA) was used at a 1:50 dilution and goat anti-mouse IgG Cy3 conjugate (Jackson Immuno Research, USA) was used as the secondary antibody (1:200). Tight junction expression of *zona occludens*-*1* (ZO-1) was measured with a mouse anti-human FITC-conjugated antibody (1:50, Invitrogen, USA). A rabbit antibody against human vimentin (1:200, Abcam, USA) was used with a FITC conjugated goat anti-rabbit antibody as a secondary antibody (1:100, Jackson ImmunoResearch, USA). Standard fixation and immunofluorescence staining protocols were performed as described in the product datasheet. The cell nuclei were stained with 4′6-diamidino-2-phenylindole (DAPI, Sigma Aldrich) for 1 min and then washed with 1X PBS.

### Membrane Permeability Assay

A 3 mL syringe was filled with a 2.5 mg/mL solution of FITC-dextran (150 kDa MW, Sigma Aldrich, USA) and mounted on a syringe pump. The syringe was connected to the top chamber with microbore tubing, while 200 µL of 1X PBS were added to the outlet reservoir and to the inlet and outlet of the bottom chamber. Perfusion was run at 2.5 µL/min. 100 µL samples were collected from each of the reservoirs and replaced with 100 µL of PBS at 1, 2 and 3 h intervals. Fluorescence intensity in the collected effluent was measured using a fluorescence microplate reader (GloMax^®^ Multimode Readers, Promega) at 470 nm excitation and 520–550 nm emission range (details in Supplemental Material).

### Effluent Collection and Protein Measurement

The devices were cultured initially for 14 days in either complete growth medium, or complete EBM-2 medium supplemented with oestradiol (1 nM) until both cell monolayers reached 80% confluence. The complete growth medium for the stromal chamber was then supplemented with MPA (0.5 nM) and media was collected and changed daily (300 µL) for an additional 14 days from both inlet and outlet. Collected effluents were then analysed by measuring prolactin production, a marker of decidualization, using an enzyme-linked immunosorbent assay (ELISA Duoset, R&D systems). The ELISA was performed according to the manufacturer’s instructions using 50 µL samples. The plate was read using an absorbance microplate reader (GloMax^®^ Multimode Readers, Promega). The results are representative of three different experiments. Due to the patient-related variability among experiments, we normalized prolactin concentration to the minimal mean prolactin production at day 2. Statistical analysis was performed by a 2 way ANOVA using a Bonferroni correction. Statistical significance was calculated as *p* < 0.05.

## Results

### Fabrication of a Dual-Chamber Microfluidic Device with a High-Resolution Porous Membrane

**Figure 3:**
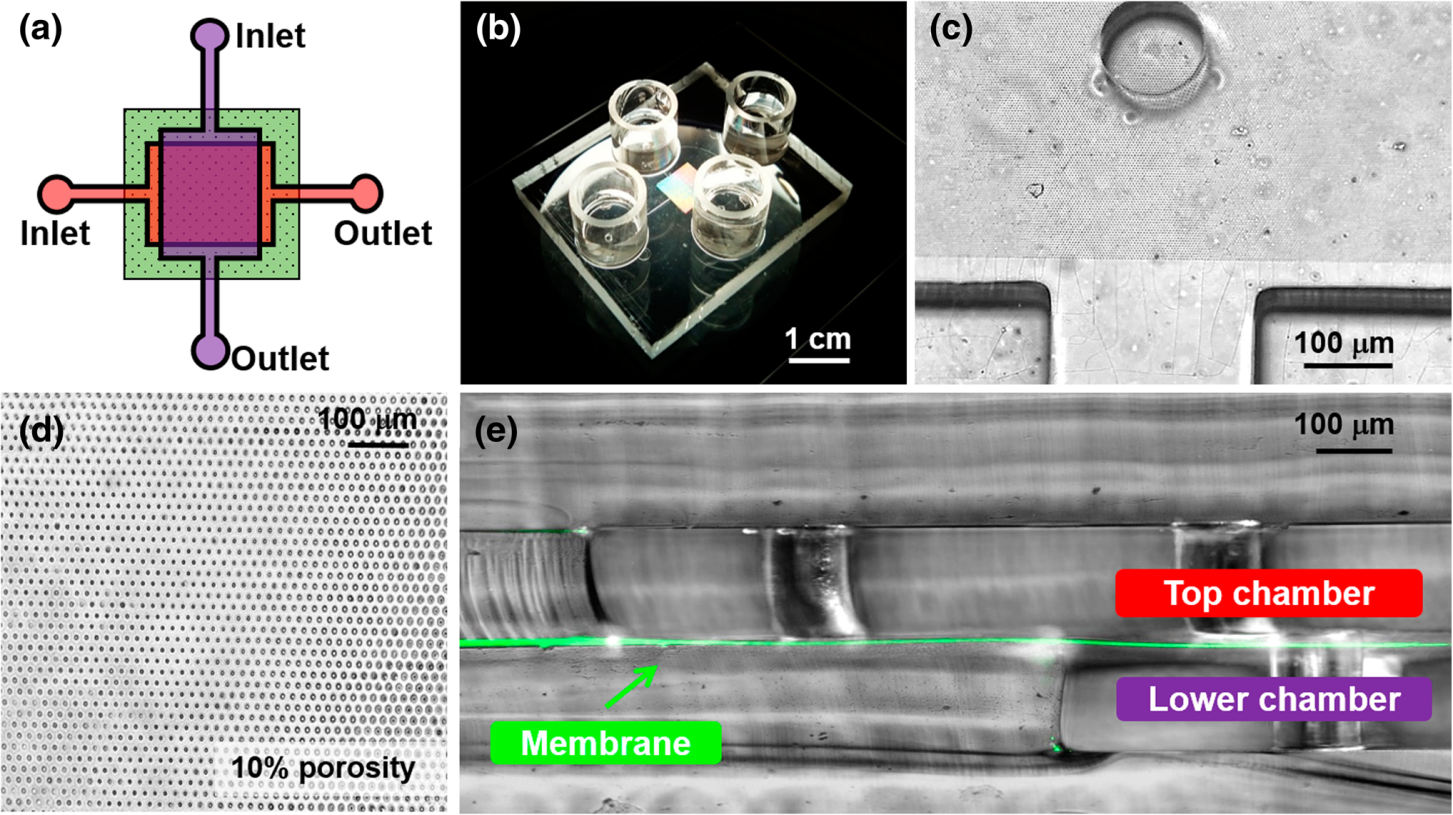
Design and characterization of the dual chamber microfluidic device with a high resolution membrane. (a) Schematic of the two chambers design. (b) A photograph of the assembled PDMS device with four reservoirs corresponding to inlets and outlets: the porous membrane appears translucent. (c) A top view of a chamber with bonded porous membrane: the border of the porous region on the top can be distinguished by the lower non-porous region, and thus easily aligned with the chamber wall and the circular pillars; one of the 5 pillars appears on the top, in the exact center of the chamber. (d) Top view image of the 1002F membrane with 10% porosity inside device. The porosity is calculated as the ratio of the combined area of the pores to the total area of the patterned region. (e) A cross section of the assembled device: the two chambers and the cylindrical pillars can be visualized as well as the green coloured 1002F membrane (enhanced background fluorescence was used to visualize the membrane in green).

As noted above, the ability of researchers to understand the intercellular paracrine interactions that regulate endometrial function within the perivascular microenvironment is limited by a lack of an appropriate model to accurately recreate the perfusion characteristics and physical compartmentalization at this tissue site. To overcome these constraints, we designed a microfluidic device and integrated a transparent semipermeable membrane (Fig. 3) to model the endometrial vascular interface between endothelial and endometrial stromal cells that mediate critical reproductive processes.

The device consists of 2 orthogonal microfluidic chambers, each one providing 29.45 mm^2^ of cell growth area and a volume of 4.7 µL. The chambers are divided by the 1002F resin-based membrane, and sealed by plasma bonding. The 1002F membrane is biocompatible, has a 6-μm thickness (DekTak3 surface profilometer) and 2-μm circular pores that allow soluble factor communication and cellular contact between cells cultured on the two sides.^29^ The patterned membrane fully spans the chamber to provide a compartmentalized region, suitable for cell co-culture assays (Fig. 3). Due to the aspect ratio of the fluidic chamber, the flexibility of the membrane and the use of automated perfusion of the endothelial chamber, we incorporated a series of pillars within the chambers that provide structural support to the membrane. While it is important to note that these pillars may alter dynamic flow profile inside the chamber, we always observed homogeneous cell distribution during loading and uniform proliferation and polarization throughout the chamber (see next sections). Furthermore, the density and size of membrane pores does not affect the cell loading and the flow dynamic inside the device. Repetitive loading of cells in the two chambers did not show leakage of cells through the pores (since the 2-μm diameter is smaller than the size of the individual cell).

The 1002F membrane is transparent and the porous region can be distinguished from non-porous one (Fig. 3c). This simplifies layer alignment during device assembly and aids in determining the cell density ratios following device seeding. Being both PDMS and 1002F membrane transparent, we are able to characterize real time cell maintenance inside the device as described in detail below and perform low background immunofluorescent imaging.

### Characterization of the Microfluidic Model of the Endometrial Perivascular Stroma

Little is known about the role of the vascular endothelium in regulating endometrial reproductive processes due to a lack of adequate models that physiologically mimic the *in vivo* characteristics of human tissue. To evaluate the cellular growth inside the device, we seeded primary human endothelial cells and endometrial stromal cells in the top and bottom chambers of the device, respectively. Both cell types were loaded simultaneously (1 × 10^6^ cells/mL), leading to a final cell seeding density of 5000 cells/mm^2^. Static culture conditions inside the OoC were maintained for up to 28 days, corresponding to the length of an idealized menstrual cycle.

**Figure 4:**
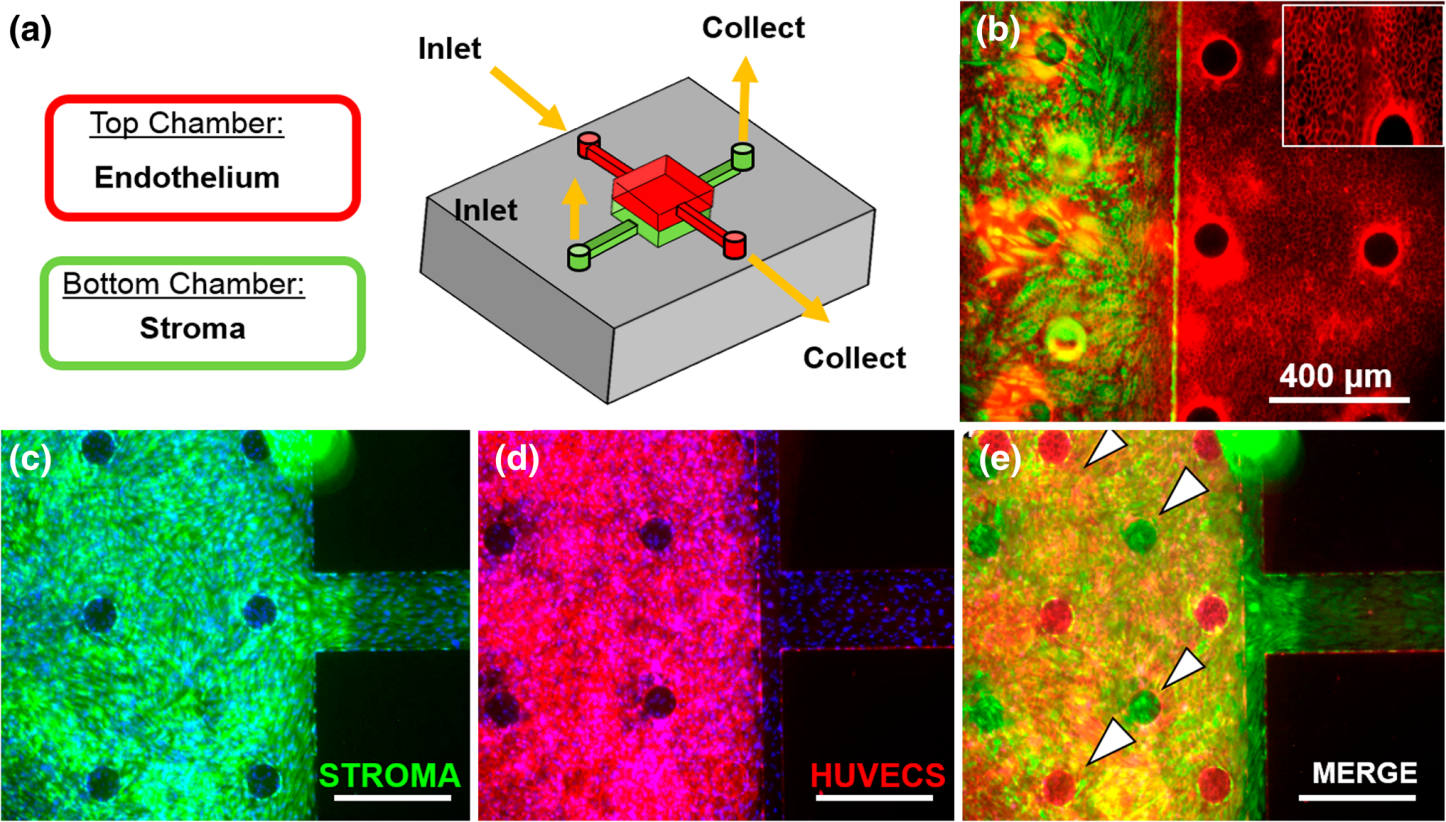
Characterization of co-culture of endometrial stromal fibroblasts (stroma) and HUVECs in the two-chamber device. (a) Schematic of the perivascular stroma model. (b) Characterization of compartmentalization and morphology: the stromal cells were cultured in the lower chamber and stained for vimentin (in green), while HUVECs were cultured in the top chamber and stained for CD31 (in red) (inset, ×100). (c) Stroma compartment showing the confluent layer of cells. (d) Endothelial compartment with confluent layer of CD31 stained HUVECs. (e) By merging the three channels (DAPI, FITC, CY5) it is possible to visualize the identity of each cell layer growing within the two compartments. As a consequence of the circular pillars (pointed by arrows), it is possible to visualize a single cell type at a time (DAPI for nuclei staining in blue, ×40 for c, d, e). Scale bars = 400 μm.

In order to confirm the compartmentalization of the two cell types, the vascular monolayer was selectively stained for the endothelial cell marker CD31, while the stromal compartment was stained for vimentin (Figs. 4b and 4c). We observed a clear compartmentalization of the two cell types throughout the device. Each cell type was observed to adopt characteristic morphologies similar to those seen in cells cultured on traditional polystyrene cell culture dishes, with distinct cobblestone morphology exhibited by the endothelial monolayer and a confluent layer of the stroma fibroblasts that initially exhibited a striated morphology prior to decidualization (Fig. S2). Furthermore, there were no distinguishable morphological differences in the endothelial layer between porous and non-porous regions of the membrane and was confirmed by CD31 immunofluorescent staining (Fig. S1a). Over time, endothelial cells were able to colonize and establish a confluent layer at the top and at the bottom of the chamber (Fig. S2d).

### Validation of the Endothelial Monolayer Shear Stress Polarization, Actin Cytoskeleton Realignment and Barrier Function

**Figure 5:**
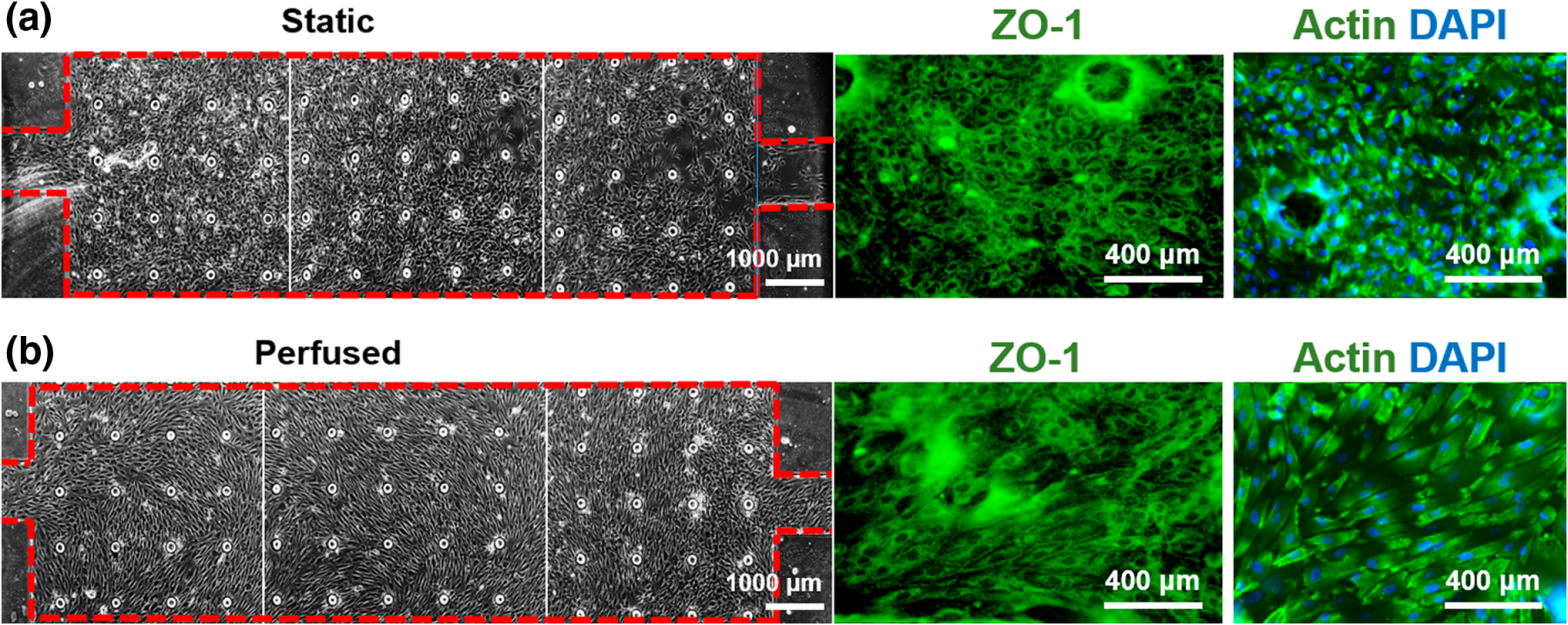
Wall shear stress induces polarization of endothelial cells inside the device. (a) Without shear stress, cells showed a non-directional and disorganized cytoskeleton. (b) In the presence of flow, cells elongated and aligned with the flow (from left to right). Right panels show ZO-1 (green) and F-actin filaments staining (green with blue nuclei stained in blue for contrast) in the two conditions (Bright field images at ×40, fluorescent images at ×100).

Vascular blood flow is an essential characteristic of endothelial cell biology and thus in order to appropriately mimic the fluid dynamic condition of the perivascular endometrium within our OoC, only the endothelial cells were dynamically perfused, while the stroma cells were cultured under static conditions. We utilized morphological polarization as a marker of culture conditions exhibiting physiologic shear stress. The endothelial cells were allowed to proliferate to confluence in full medium in static conditions (Fig. 5a) and subsequently exposed to 1 μL/min flow for a minimum of 4 days (Fig. 5b). The stroma were kept in static conditions since this cell type is not affected by direct shear stress in physiological conditions within the endometrium.

**Figure 6:**
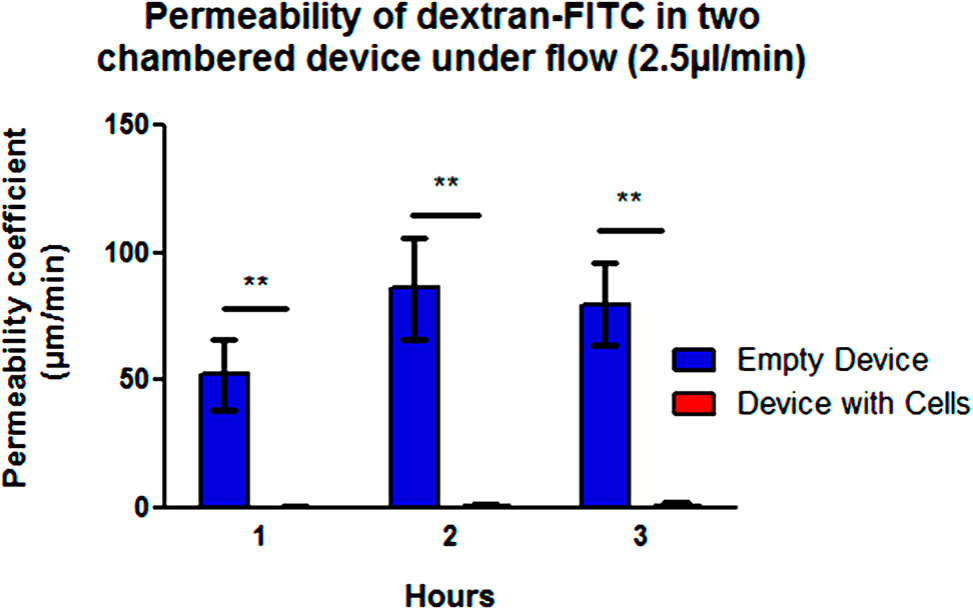
Permeability coefficient of membrane in two-chamber platform calculated using macromolecule diffusion (FITC-dextran) shows endothelial cells making a tight barrier, (*p* = 0.0098).

Tight junction formation, observed by staining ZO-1, was positively identified in both conditions, but only the shear stressed monolayers formed clear parallel margins around the cell body. These cells showed polarization and changes in cell shape from polygonal to ellipsoidal (Fig. 5b), as result of alterations in the F-actin filaments and reorientation of stress fibers in the cytoskeletal structure. As a proof of principle, long term culture of a confluent endothelial layer was maintained under continuous perfusion for more than 24 days (Supplemental Material). The dual-chamber device also permits assaying the functional integrity of the endothelial monolayer in forming a barrier to diffusive transport. We evaluated the permeability of the endothelial monolayer by measuring the transfer of fluorescently labelled dextran (150 kD MW) between the two chambers of the device. Compared to an unpopulated device, there was a significant reduction in the permeability coefficient of the membrane once the endothelial layer was confluent (Fig. 6). These findings confirm both the permeability of the membrane and the establishment of a confluent and tight endothelial monolayer working as barrier to macromolecule transport. The comparison of single cultures of stromal cells in the bottom chamber, compared to co-culture of both endothelial and stromal cells, suggests that it is the endothelium that is promoting the barrier capabilities (Fig. S3).

### Validation of Reproductive Function of the Endometrial Perivascular Specialized Stromal Fibroblasts

**Figure 7:**
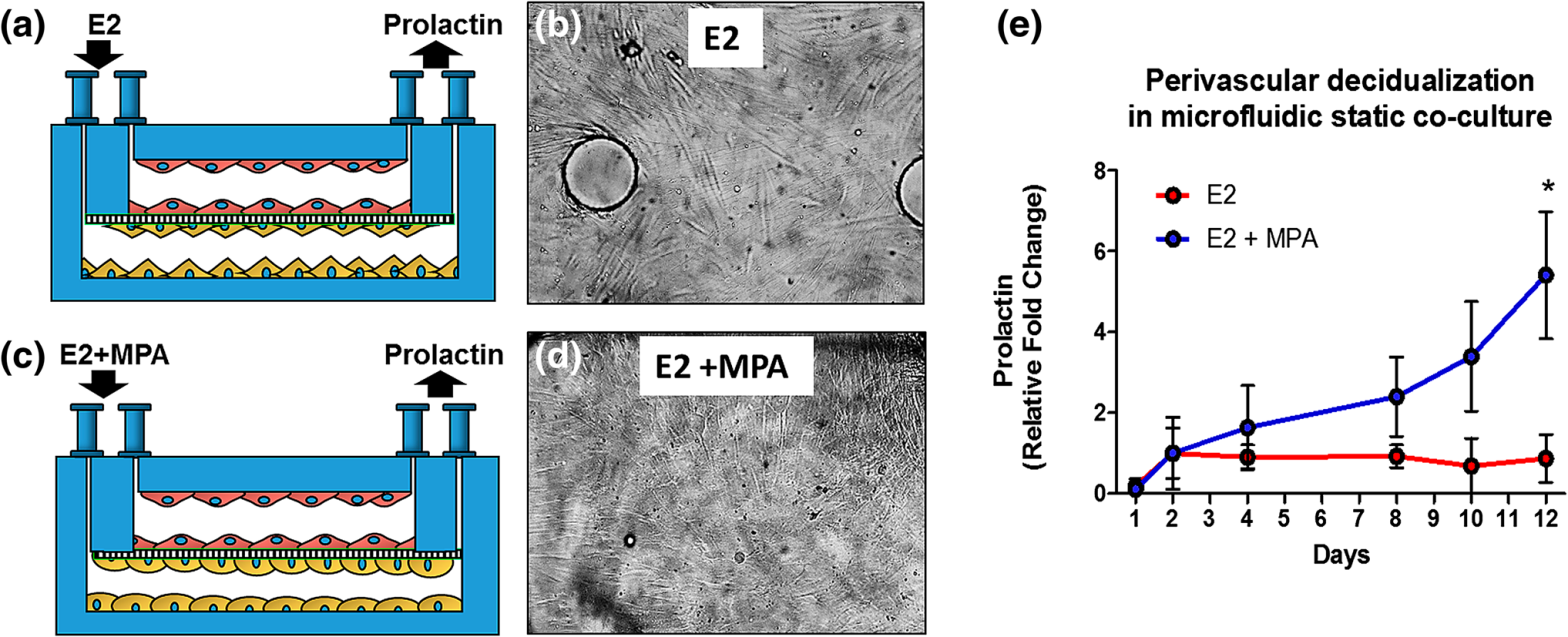
Validation of physiological response of the perivascular stroma to endocrine cues. (a) Schematic of the experimental design of the co-culture device. (b) Bright field image of co-culture with stromal cells directly cultured with oestradiol (E2) alone for 14 days. (c) Schematic of the stimulation settings of the co-culture device with E2 and MPA with the corresponding changes of morphology for the stroma from fibroblast-like to cuboidal shape, characteristic of the decidualized cells. (d) Bright field image with stroma cells cultured with E2 + MPA for additional 14 days shows morphological changes (×10). (e) Prolactin (PRL) production measured by ELISA increases over time under the influence of E2 + MPA, but not when cultured with E2 only (*p* < 0.05).

Endometrial stromal fibroblasts are specialized cells, uniquely different from other types of mesenchymal cells in the human body. Reflecting their fundamental role in the endocrine-mediated function of the endometrium, they express both oestrogen and progesterone receptors and are highly responsive to the ovarian sex steroids changes across the menstrual cycle.^16^ Under the influence of progesterone during the secretory phase of the menstrual cycle, stromal fibroblasts begin the process of decidualization. In seminal histologic papers, Noyes *et al.* first identified the process of decidualization as morphological changes in stromal cells from the typical spindly shaped fibroblast to a cuboidal and glycogen rich decidual cells.^39^ In addition, biochemical changes within the differentiating stromal cells include the active secretion of multiple progesterone-induced proteins that accurately serve as biomarkers of the decidualization process.^7^ Therefore, to validate both the physiological function and the sensitivity of our microfluidic perivascular stroma co-culture model, we measured the capability of the stromal cells to decidualize when stimulated by MPA, a synthetic progestin that exhibits more stability under culture conditions than natural progesterone. An idealized, complete physiological menstrual cycle was simulated, by supplementing the cell culture medium for the first 2 weeks with oestradiol alone (as in the proliferative phase, Fig. 7a), and subsequently with oestradiol and MPA for the following 2 weeks (as in the secretory phase, Fig. 7b).

A shift in the morphology of the stromal cells toward a round and cuboidal shape was observed after 10 days of MPA treatment (Fig. 7b) as expected from other *in vitro* studies.^4^,^20^ For a quantitative analysis of biochemical markers of decidualization in response to different steroids treatments, we measured prolactin (PRL) secretion in spent media collected daily from gravity fed co-cultures using ELISA. A continuous increase of PRL over time was measured from stroma stimulated with oestradiol and MPA compared to the stroma treated only with oestradiol, which exhibited consistently low production of PRL after 2 weeks of treatment (Fig. 7a). An additional perfused device was treated with an oestradiol + MPA and 8-Br-cAMP, a potent, intracellular driver of stromal decidualization that is frequently utilized for *in vitro* studies of progesterone action in endometrial cells and cell lines.^7^ As expected, we observed significantly higher PRL production from cultures exposed to 8-Br-cAMP, approximately one order of magnitude higher after 4 days of treatment (Fig. S4). These findings additionally demonstrate the utility of our device to discriminate changes in the rate of *in vitro* decidualization between cultures exposed to MPA alone vs. culture exposed to MPA plus cAMP. Discriminating among biological agents that impact endometrial decidualization at the perivascular interface will be important for future studies using our OoC device to identify potential agents that influence progesterone action within the endometrium. Importantly, our results equally demonstrate the capability of the model to mimic each phase of a full 28-day menstrual cycle, with continuous sampling capabilities for quantitative biochemical analysis.

## Discussion

In this work, a novel resin based high-resolution porous membrane was used to design and fabricate a dual-chamber microfluidic model. The porous part of this thin membrane is macroscopically translucent which represents a favourable aspect in the assembly and alignment stages of the microfluidic chambers impressed in the PDMS layers. Moreover, being that the remainder of the membrane is not porous, bonding by plasma activation is optimized and leakage is prevented. This unique membrane represents an interesting alternative to opaque polycarbonate etched membranes, thicker PDMS based membranes^26^ or more fragile vitrified membranes.^36^

We adopted the microfluidic approach to develop a novel, physiologically relevant, OoC model of the endometrial perivascular stroma. Within our model, for the first time to our knowledge, primary human endothelial and endometrial stromal cells were co-cultured in communicating chambers and maintained for 28 days, spanning the physiological human menstrual cycle duration. Our OoC model will provide an *in vivo*-like human model that avoids the expense of primate models or the use of small animal models that are often biologically distinct and less clinically relevant.^42^

Biological responses to endocrine signalling were examined in this paper by mimicking the oestrogen and progesterone changes that accompany the transition from proliferative to secretory phase *in vivo*. We measured the functional capability of the perivascular stroma to undergo decidualization. Early evidence of stromal/decidual transformation was observed after 10 days of combined oestrogen and progestin treatment in the OoC. This transformation can be utilized in future studies by us or others as an established marker of endometrial viability under controlled conditions. Clinically, our OoC model may also have relevance since similar histological changes have been observed *in vivo* after hormone therapy for prevention of post-menopausal morbidity or treatment of endometrial hyperplasia and carcinoma.^12^

Importantly, this co-culture system allows simultaneous analysis of both stromal decidualization and endometrial vascular function under controlled physiological conditions, such as endothelial cells remodelling and vascular barrier formation. The device provides sufficient sensitivity for assessment of biochemical changes that also reflect phenotypic changes. Together, these findings validate the ability of the microfluidic model to recapitulate and examine a physiological reproductive process. These experiments position our model as a novel method to determine the normal homeostatic function of the vascular system in the endometrium under hormonal regulation. While beyond the scope of this report, the model can subsequently be used by us and others to begin to dissect key elements of endothelial-stromal crosstalk and the impact of this communication on inflammation and disease-related reproductive processes such as endometriosis.^38^ In this regard, our OoC model as designed to control perfusion of the endothelial compartment and eventually allow the introduction of immune cells into the microfluidic co-culture device to establish a model of peripheral leukocyte recruitment into the endometrium. Specifically, in the absence of pregnancy, ovarian progesterone levels decline, triggering a local inflammatory response involving a vast infiltration of leukocytes, release of cytokines, and activation of matrix metalloproteinases.^27^ The vascular endothelium act as the interface between peripheral circulating immune cells and endometrial tissue. Hence the loss of anti-inflammatory action of progesterone during the latter part of the secretory phase is both directly and indirectly linked to phenotypic changes within the endothelium that affect immune cell migration.^34^,^43^ Thus our OoC model is compatible for future studies of cell interaction during menstruation and in inflammatory endometrial diseases (i.e. stroma, endothelial and leukocytes).

Noteworthy, PDMS has an ability to absorb hydrophobic molecules, specifically hormones. However, until materials with improved absorption behaviour become available, PDMS remains the most commonly used and accepted material for prototyping biomedical microfabrication technologies. The presented results replicate previous observations in traditional *in vitro* polystyrene culture dishes in terms of hormone responsive biochemical and morphological decidualization, suggesting that absorption of steroids by the membrane was not an impediment to cellular response.

Modelling the human endometrial microenvironment at the immuno-endocrine inflammatory axis offers the capability to better understand complex biological and pathogenic factors that act to support or disrupt endometrial function. Since decidualization is a critical process for embryo implantation, the ability to explore the earliest stages of decidualization will provide a unique screening tool for fertility studies. Finally, our OoC model of the endometrial perivascular stroma lends itself for the screening of pharmaceutical agents or environmental toxicants that may alter reproductive health or promote reproductive dysfunctions.

## Electronic supplementary material

Below is the link to the electronic supplementary material.

Supplementary material 1 (PDF 942 kb)
