# Supplementary material for: Compartmentalized Culture of Perivascular Stroma and Endothelial Cells in a Microfluidic Model of the Human Endometrium
Source: Ann Biomed Eng. 2017 Jan 20;45(7):1758–69. doi: 10.1007/s10439-017-1797-5 (PMC5489603; doi:10.1007/s10439-017-1797-5)
Supplement: Supplementary file 1 — Supplementary material 1 (PDF 942 kb) [file 10439_2017_1797_MOESM1_ESM.pdf]

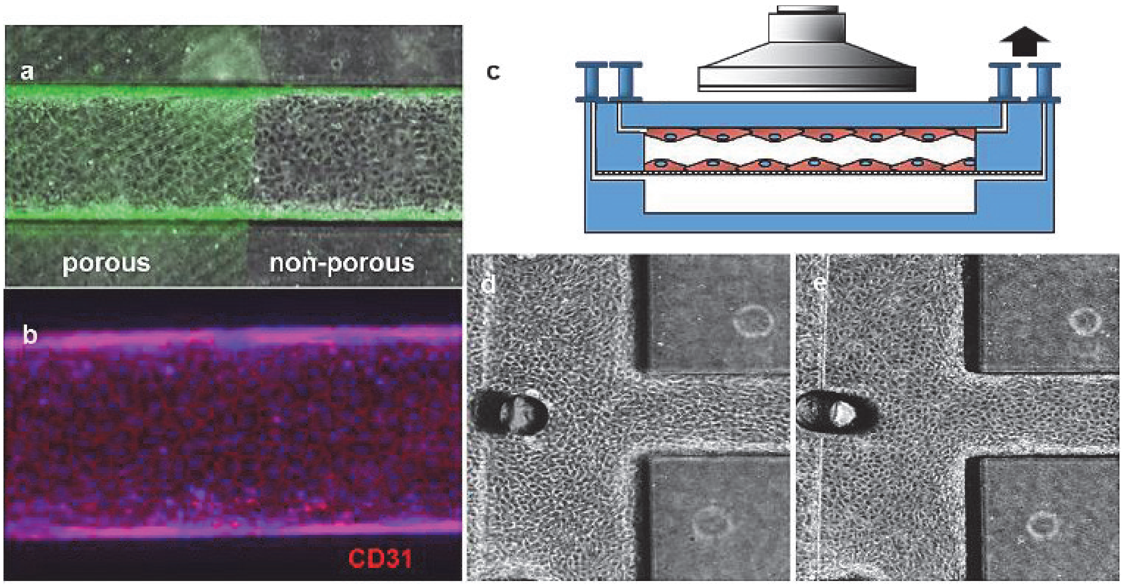

Figure s1. Endothelial growth inside two chambers device on porous membrane. (a) Vimentin staining of stroma cultured on the 1002F membrane inside the microfluidic chamber: morphology and density of cells are not affected by the presence of pores in the substrate (10x). (b) CD31 staining of HUVECs shows a confluent and tight cell layer (10x). (c) A schematic of the microfluidic device loaded with HUVECs in the top chamber: the cells form two confluent layers, one on top and one at the bottom of the chamber. (d-e) By focusing at different z-levels, corresponding to top and base of the circular supporting pillar, it is possible to focus on the two confluent layers inside the chamber.

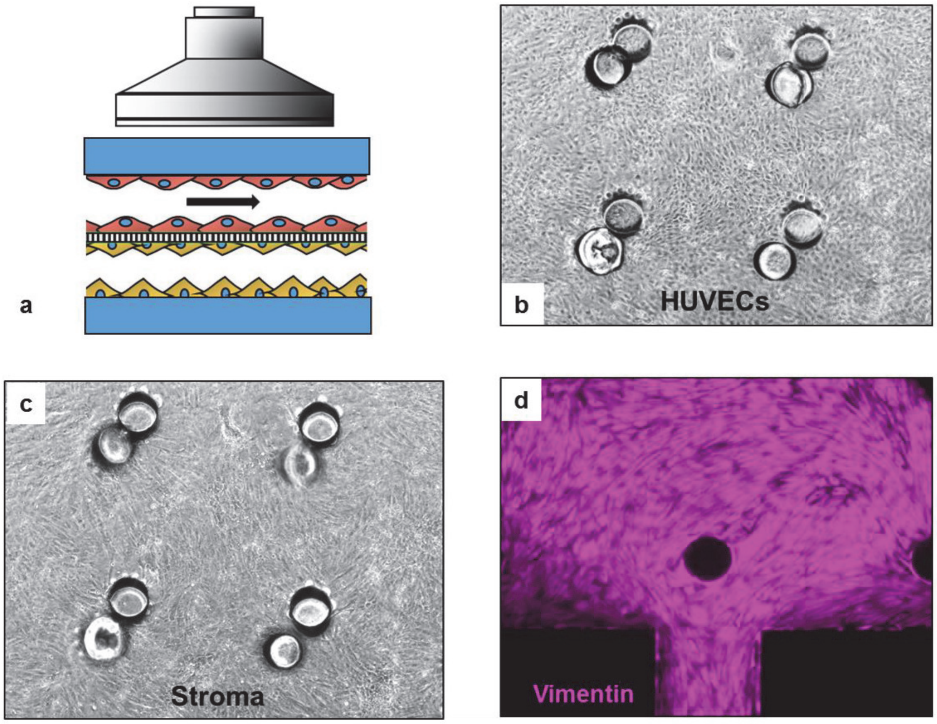

Figure s2. Co-culture of stromal and endothelial cells inside device. (a) Schematic of the co-culture protocol for shear stress experiments. The stromal cells are cultured in static conditions, while continuous laminar perfusion is maintained on endothelial chamber. (b-c). Brightfield images of the region of co-culture inside dual chamber device under static conditions identify two monolayers of each cell type. (d). Stromal compartments stained for vimentin demonstrate a confluent monolayer stromal fibroblasts (40X).

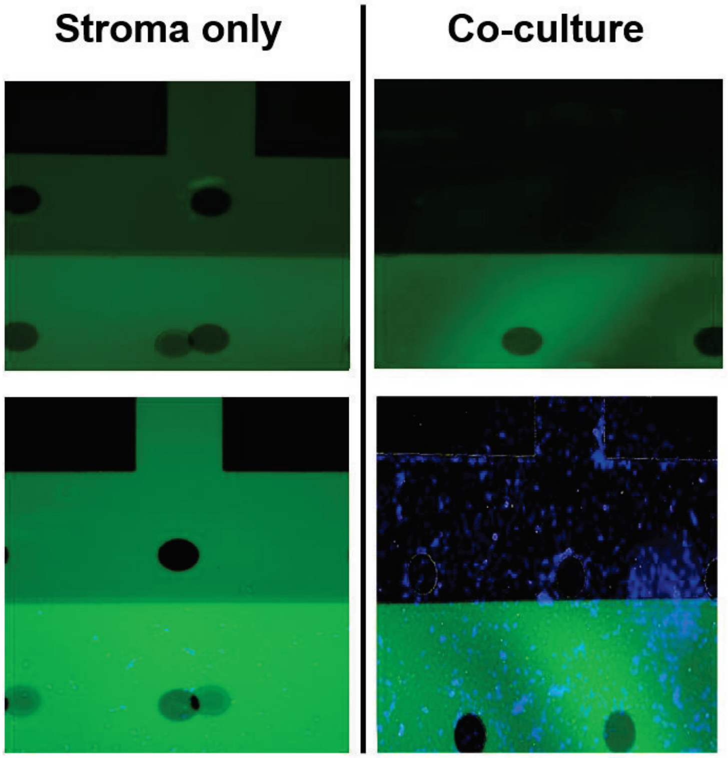

Figure S3. The permeability of the dual chamber device was assessed by flowing FITC dextran through the device, for 2 hours. In this figure the green fluorescent dextran crosses the permeable membrane when only the stroma cells are cultured in the device (left column). When both cell types are cultured in the system, and once the endothelial cells form a tight layer, the FITC dextran did not cross the membrane and was confined in the endothelial top chamber. The permeability was quantified by measuring the intensity of the fluorescent in the different samples collected form the two compartments. Knowing the concentration of FITC-dextran in the initial solution (50mg/mL), we first generated a standard curve with a serial dilution of the solute for calibration and to correlate the measured fluorescent intensity with the concentration. The amount of transported solute (ΔQ) was obtained by summing the transported solute mass over time. The permeability coefficient was calculated using the equation Pcoeff= (ΔQ/ΔT)/(A × Co), where ΔQ/ΔT is the slope of the linear portion of the compound transported vs. time curve, A is the area of the membrane, and Co the initial concentration of FITC-dextran (mg/mL) (47). This simplified equation was obtained considering the diffusion of solute negligible when compared to bulk flow of fluid across the hydrophilic membrane, with 2 μm pores, not covered by the cells. The solute reflection coefficient, σ, was as well assumed ~0, considering the large pores and the hydrophilic properties of the membrane once equilibrated with full medium. Statistical analysis was performed using a Student t-test of 3 different empty devices against 3 different co-cultures.

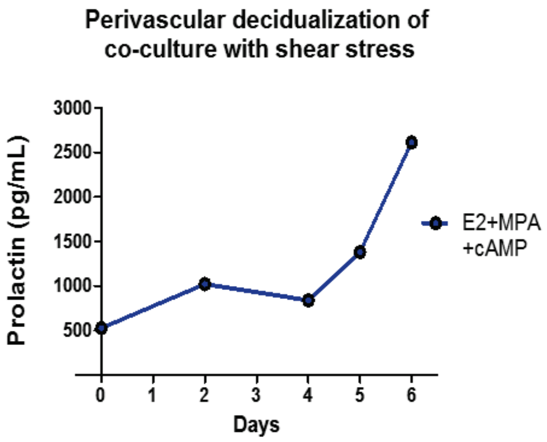

Figure S4. Validation of physiological response of the perivascular stroma to endocrine cues. PRL production measured by ELISA increases over time under the influence of E2 + MPA and cAMP (0.5nM).
